# Evaluation of the IAEA‐TRS 483 protocol for the dosimetry of small fields (square and stereotactic cones) using multiple detectors

**DOI:** 10.1002/acm2.12792

**Published:** 2019-12-30

**Authors:** Clare L. Smith, Atousa Montesari, Christopher P. Oliver, Duncan J. Butler

**Affiliations:** ^1^ School of Health and Biomedical Sciences RMIT University Bundoora VIC Australia; ^2^ Peter MacCallum Cancer Centre Melbourne VIC Australia; ^3^ Australian Radiation Protection and Nuclear Safety Agency Yallambie VIC Australia

**Keywords:** output factors, small field dosimetry, stereotactic cones, TRS‐483

## Abstract

The IAEA TRS 483 protocol^1^ for the dosimetry of small static fields in radiotherapy was used to calculate output factors for the Elekta Synergy linac at the Australian Radiation Protection and Nuclear Safety Agency (ARPANSA). Small field output factors for both square and circular fields were measured using nine different detectors. The “corrected” output factors (ratio of detector readings multiplied by the appropriate correction factor from the protocol) showed better consistency compared to the “uncorrected” output factors (ratio of detector readings only), with the relative standard deviation decreasing by approximately 1% after the application of the relevant correction factors. Comparisons relative to an arbitrarily chosen PTW 60019 microDiamond detector showed a reduction of maximal variation for the corrected values of approximately 3%. A full uncertainty budget was prepared to analyze the consistency of the output factors. Agreement within uncertainties between all detectors and field sizes was found, except for the 15 mm circular field. The results of this study show that the application of IAEA TRS 483^1^ when measuring small fields will improve the consistency of small field measurements when using multiple detectors contained within the protocol.

## 1 INTRODUCTION

The release of the first international code of practice for the dosimetry of small static fields IAEA TRS 483^1^ was derived by an international working group in collaboration with the American Association of Physicists in Medicine. It aims to deliver worldwide consistency in small field reference dosimetry for clinical radiotherapy, which is traceable to a primary standard. The protocol details the methods and corrections to be applied to various detectors for determining small field output factors, as well as machine specific details.

The protocol was needed due to the increasing clinical use of stereotactic radiotherapy treatments such as Stereotactic Ablative Body Radiotherapy for the targeting of small tumors with single high‐dose fractions.^2^, ^3^, ^4^, ^5^ The selection of detectors and methods used for the dosimetry of these beams are important for both clinical quality assurance and patient safety during treatment.

Small field dosimetry is challenging, particularly for very small fields due to inherent issues that affect the measured output factors such as steep dose gradients and partial occlusion of the radiation source.^3^, ^6^, ^7^, ^8^, ^9^, ^10^ Furthermore, it usually requires a detector with a small active volume and high spatial resolution.^3^, ^8^, ^11^ Detector‐specific issues including a lack of lateral electronic equilibrium, dose averaging, and nontissue equivalence need to be considered when choosing an appropriate detector.^3^, ^10^, ^11^

Attempts to consolidate other studies to provide detector correction factors in small fields are difficult due to differences in the how field size was defined, depth and source to surface distances (SSDs) and the size of the reference field.^1^ At the time of completing this work there was only one other study,^11^ which we were aware of, that had examined the protocol’s correction factors for the IBA‐SFD detector in circular fields (5–40 mm). A separate study had examined six of the detectors listed in the protocol,^12^and determined large differences between detectors for the uncorrected output factors when using smaller field and cone sizes, with shielded diodes having higher uncertainties due to their construction.

In this work output factors were measured using nine different detectors included in the IAEA TRS 483 protocol. The corrected and uncorrected output factors were compared for five stereotactic cones (nominal 5–50 mm) and seven square fields (1 cm × 1 cm to 6 cm × 6 cm). To investigate the potential variations within detectors of the same type, the output factors of the stereotactic cones were measured using four different PTW 60019 microDiamond detectors. An uncertainty budget was completed for all detectors and relevant field and cone sizes examined.

## 2 MATERIALS AND METHODS

### 2.1 Square field measurements

The Elekta Synergy linear accelerator is equipped with a multi‐leaf collimator (MLC) consisting of 80 leaves, with a projection of 10 mm at the isocenter. Output factors in field sizes of 1 cm × 1 cm, 1.5 cm × 1.5 cm, 2 cm × 2 cm, 2.5 cm × 2.5 cm, 3 cm × 3 cm, 4 cm × 4 cm and 6 cm × 6 cm (defined by the MLC) were measured, referenced to a 10 cm × 10 cm reference field.

### 2.2 Stereotactic conical collimator system

**Figure 1:**
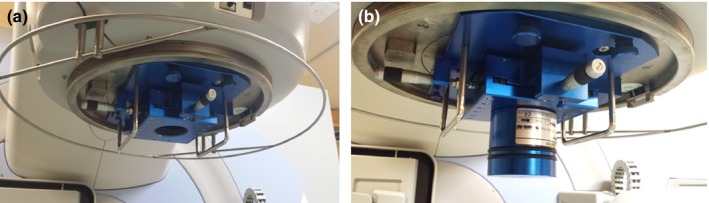
The cone attachment plate (colored blue) mounted on the linac head (a) and with the cone attached (b).

The Elekta stereotactic conical collimator system consists of five circular cones of varying diameter, which attach via a plate to the linac head (Fig. 1).

The output factors in fields defined by circular cones of nominal diameters at the isocenter of 5, 7.5, 10, 15 and 50 mm were measured and referenced to MLC defined 3 cm × 3 cm (for 5–15 mm cones) and 6 cm × 6 cm fields (for the 50 mm cone). The cone axis was aligned to the central axis of the beam and was consistent for all measurements.

### 2.3 Detectors

Nine different detector types were investigated with their active volumes and description presented in Table 1. The detectors are classed into one of four categories based on the TRS‐483 protocol; unshielded diodes and microDiamond, shielded diodes, micro ionization chambers and mini ionization chambers.

**Table 1:** The nine different detector types investigated.

Detector	Detector type (TRS‐483 Table 37)	Abbrev. used	Active material	Active volume
microDiamond PTW 60019	Unshielded & PTW 60019 microDiamond	microD	Synthetic single crystal diamond	0.004 mm^3^
IBA‐EFD3G	As above	EFD	Unshielded p‐type silicon diode	~0.19 mm^3^
SRS diode PTW 60018	As above	SRS	Unshielded p‐type silicon diode	0.3 mm^3^
Electron diode PTW 60017	As above	E 60017	Unshielded p‐type silicon diode	0.03 mm^3^
Photon diode PTW 60016	Shielded diodes	P 60016	Shielded p‐type silicon diode	0.03 mm^3^
IBA‐PFD3G	As above	PFD	Shielded p‐type silicon diode	~0.19 mm^3^
Pinpoint PTW 31014	Micro IC	PP 31014	PMMA‐walled ionization chamber, vented to air with aluminum (99.98% purity) electrode	0.015 cm^3^
Pinpoint 3D PTW 31016	As above	PP 31016	PMMA‐walled ionization chamber, vented to air with aluminum (99.98% purity) electrode	0.016 cm^3^
Wellhöfer/IBA CC 04	Mini IC	CC04	C552‐walled ionization chamber, vented to air with air equivalent plastic electrode	0.04 cm^3^

### 2.4 Measurements

**Figure 2:**
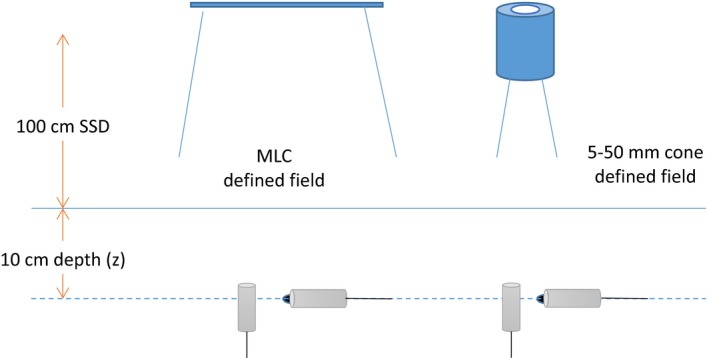
The detector setup and orientations (ionization chambers PTW 31014 and 31016, and IBA CC04 are placed horizontally, and all others vertically) as used for cone and square field measurements.

All measurements were completed using the IBA Dosimetry Blue Phantom2 tank with OmniPro‐Accept software, scanning volume of 480 mm (l) × 480 mm (w) × 410 mm (h) and positional reproducibility of ±0.1 mm.^13^ The output factor and profiles were measured at an SSD of 100 cm and water phantom depth of 10 cm (Fig. 2). Each detector was positioned at the isocenter and centered on the radiation beam using in‐line and cross‐line scans to yield the maximum signal intensity. In this work the “daisy‐chaining” method^14^ was not used.

Doses of 100 monitor units (MU) with 6 MV X‐ray beam (WFF) were delivered until three consistent measurements were recorded. A second set of measurements was completed on a different day and the average “corrected” (using factors from the protocol) and “uncorrected” output factors values were obtained as defined in Eqs. (1) and (2). While the nominal field sizes are stated in this work the field sizes were measured using in‐line and cross‐line scans, and used to determine the correction factors to be applied.

(1) $$\begin{array}{cc} \text{Corrected OF}= & \frac{\text{Detector reading},\;\text{cone or small field}}{\text{Detector reading},\;\text{reference field}}\\ & \times k\left(\text{TRS}-483:\text{Table}\;26\right) \end{array}$$

(2) $$\text{Uncorrected OF}=\frac{\text{Detector reading},\;\text{cone or small field}}{\text{Detector reading},\;\text{reference field}}$$

The relative standard deviation (RSD) for circular and square field output factors (corrected and uncorrected, all detectors) was calculated, and the percentage differences. As well, the corrected output factors for four PTW 60019 microDiamond detectors were compared for five stereotactic cone fields. Again, six output measurements were made (three measurements from two separate days) for each detector and cone size, and the average used. Finally, sources of uncertainty and their magnitude were identified and a small field output factor uncertainty budget prepared, which includes type A and B components.

## 3 RESULTS

### 3.1 Corrected and uncorrected output factor values

**Figure 3:**
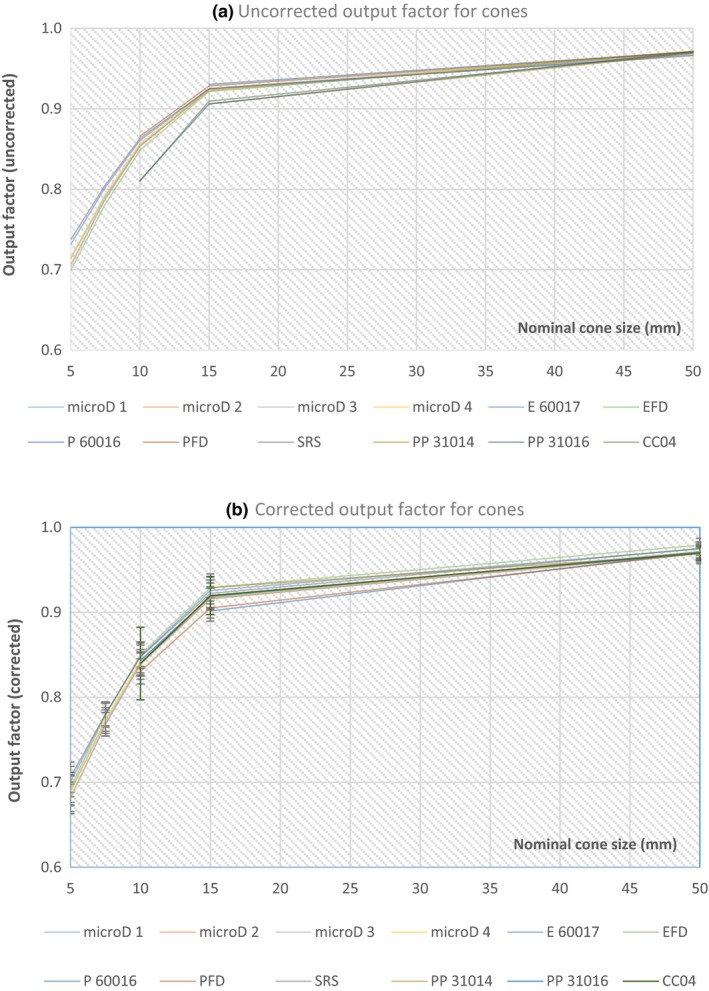
The uncorrected (a) and corrected (b) output factors for all detectors measured in circular fields defined by the Elekta stereotactic cones (5, 7.5, 10, 15 and 50 mm) relative to a square 3 cm × 3 cm and 6 cm × 6 cm fields (50 mm cone only).

**Figure 4:**
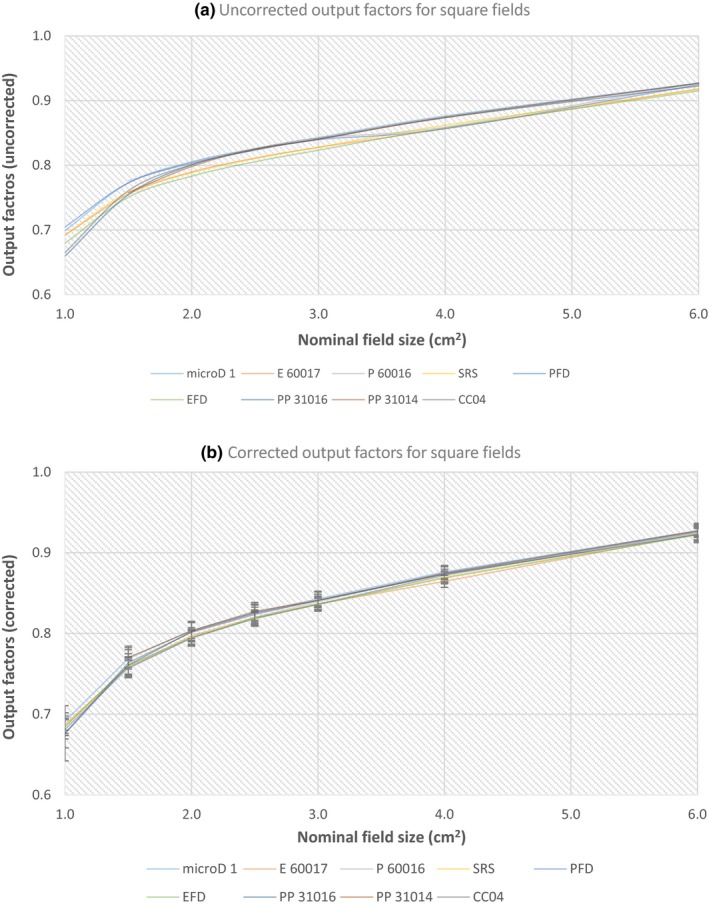
The uncorrected (a) and corrected (b) output factor values for all detectors investigated using square fields, relative to a 10 cm × 10 cm field.

The corrected and uncorrected output factor values for each detector are plotted against the nominal cone size (5, 7.5, 10, 15 and 50 mm) (Fig. 3) and square field size (1 cm × 1 cm, 1.5 cm × 1.5 cm, 2 cm × 2 cm, 2.5 cm × 2.5 cm, 3 cm × 3 cm, 4 cm × 4 cm and 6 cm × 6 cm) (Fig. 4) relative to the reference fields used (see Materials and methods). The error bars shown on the corrected figures [Figs. 3(b) and 4(b)] were determined from the uncertainty budget and are explained in Section 3 E. microD 1 – microD 4 are four different PTW microDiamond detectors with the other detectors described by their abbreviations used in Table 1, and an intercomparison of their measured output factors was completed for the circular fields only.

### 3.2 Relative standard deviation (RSD)

The RSD for circular and square field output factors (corrected and uncorrected, all detectors) was determined, including the percentage difference with and without correction (Tables 2 and 3).

**Table 2:** The RSD (corrected and uncorrected) in output factor for the stereotactic cone defined fields for 12 detectors.

Cone	RSD		
mm	Corrected	Uncorr.	Difference
5	1.2%	1.8%	0.6%
7.5	0.7%	1.0%	0.3%
10	0.6%	2.3%	1.7%
15	0.9%	0.9%	0.0%
50	0.3%	0.2%	−0.1%

RSD, relative standard deviation.

**Table 3:** The RSD (corrected and uncorrected) in output factors for the MLC defined square fields for nine detectors.

Field	RSD		
cm^2^	Corrected	Uncorr.	Difference
1.0	0.8%	2.3%	1.6%
1.5	0.6%	1.2%	0.6%
2.0	0.5%	1.0%	0.5%
2.5	0.4%	0.9%	0.5%
3.0	0.3%	0.9%	0.6%
4.0	0.4%	1.0%	0.6%
6.0	0.2%	0.5%	0.3%

RSD, relative standard deviation; MLC, multi‐leaf collimator.

### 3.3 Comparison with the ARPANSA PTW 60019 microDiamond detector (microD 1)

**Figure 5:**
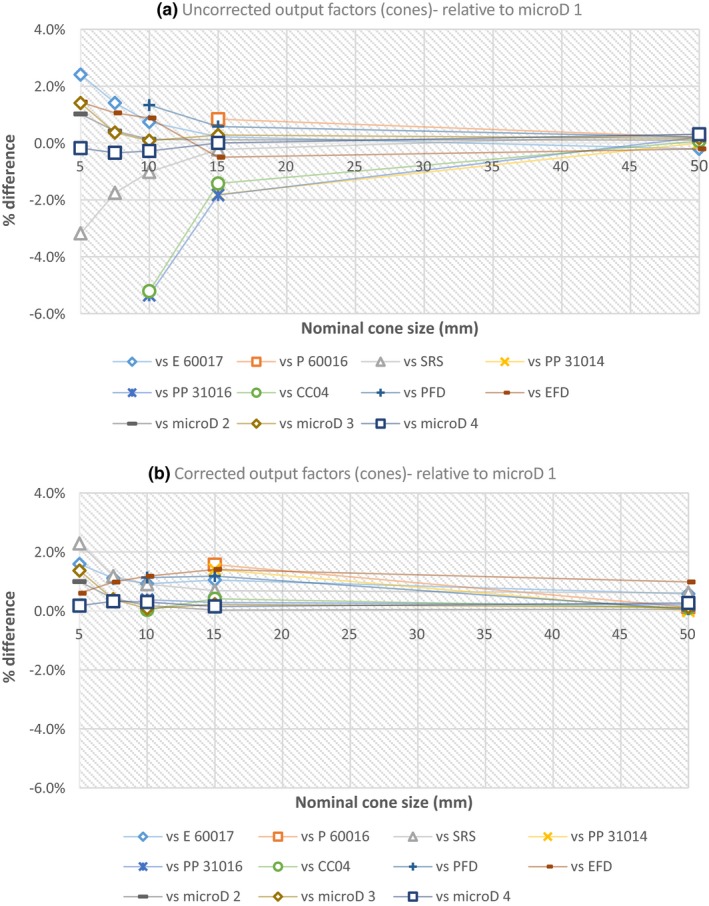
The percentage differences between the uncorrected (a) and corrected (b) output factors for each detector relative to the ARPANSA PTW 60019 microDiamond (microD 1) detector for circular fields.

**Figure 6:**
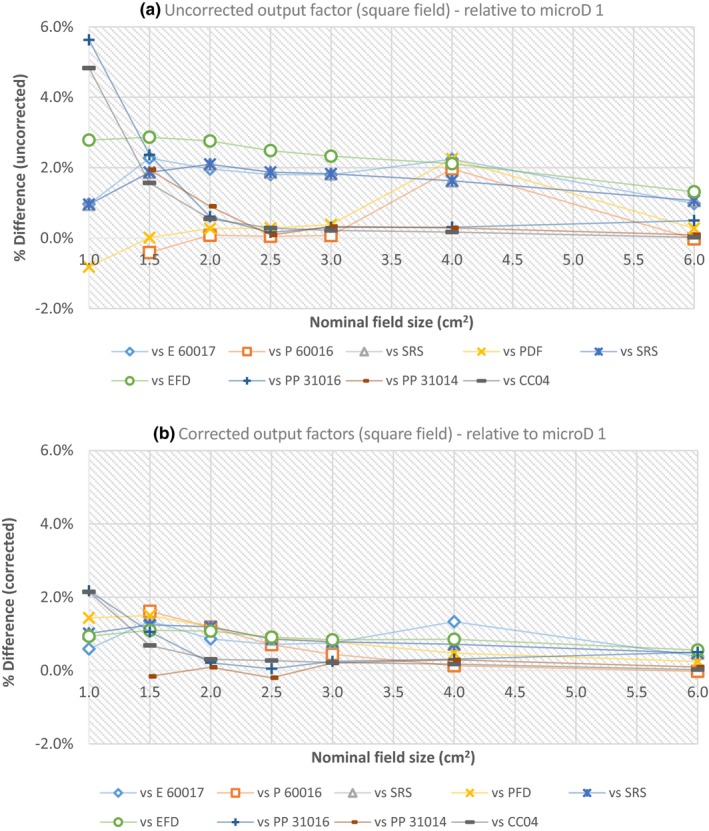
The percentage differences between the uncorrected (a) and corrected (b) output factor values for each detector relative to the ARPANSA PTW 60019 microDiamond (microD 1) detector for square field measurements.

To aid in the visual differentiation of the variation in measured output factors, all output factors have been plotted relative to a PTW 60019 microDiamond (microD 1). The uncorrected and corrected output factors are shown in Fig. 5 (circular fields) and Fig. 6 (square fields).

### 3.4 PTW 60019 microDiamond intracomparison in the stereotactic cone defined corrected output factor values

**Figure 7:**
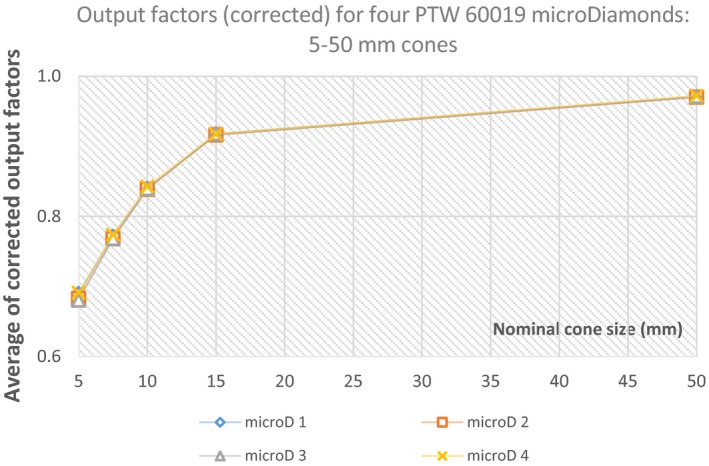
The average “corrected” output factors for the four PTW 60019 microDiamond (microD 1‐4) detectors in the five circular fields defined using the 5–50 mm cones, relative to a 3 cm × 3 cm and 6 cm × 6 cm field (50 mm cone only).

The corrected output factors for four PTW 60019 microDiamond detectors were compared for the five stereotactic cone fields. Six corrected output measurements (three each from two separate days) for each detector were completed for all cone sizes and the average used (Fig. 7, Table 4).

**Table 4:** Comparisons of the corrected output factors for the four PTW 60019 microDiamond detectors measured in the stereotactic cone fields.

Cone (mm)	Average (for 4 microD detectors)	Difference (max‐min)	Std dev.	RSD
5	0.69	1.4%	0.005	0.7%
7.5	0.77	0.4%	0.002	0.3%
10	0.84	0.2%	0.002	0.2%
15	0.92	0.2%	0.001	0.1%
50	0.97	0.1%	0.001	0.1%

### 3.5 The small field output factor uncertainty budget

Sources of uncertainty and their magnitude were identified, and a small field output factor uncertainty budget was prepared showing the combined relative standard uncertainty for circular and square fields for each detector type investigated. The uncertainty budget includes type A and B components, with data from the ARPANSA Australian Clinical Dosimetry Service (ACDS) used to determine some of the type A values.^15^ Detectors are divided into four classes as defined in the TRS‐483 protocol; microDiamond/unshielded diodes, shielded diodes, micro‐ionization chambers (Micro‐IC) and mini‐ionization chambers (Mini‐IC) (Table 1). The uncertainties in the measurements for the reference fields (cone fields; 3 cm × 3 cm and 6 cm × 6 cm, square fields; 10 cm × 10 cm) and circular and square field measurements are presented and summated in quadrature (Tables 5 and 6), according to the GUM.^16^ Explanations for the terms used and how they were determined are presented after the tables. The error bars shown in [Figs 3(b) and 4(b)] are the expanded uncertainty (k = 2).

**Table 5:** The relative standard (RS) uncertainty for all components (A and B) in the measurement of circular fields.

Quantity	50 mm cone		15 mm cone		10 mm cone		7.5 mm cone		5 mm cone	
	RS Uncert.		RS Uncert.		RS Uncert		R S Uncert.		RS Uncert.	
	100 u_iA_	100 u_iB_	100 u_iA_	100 u_iB_	100 u_iA_	100 u_iB_	100 u_iA_	100 u_iB_	100 u_iA_	100 u_iB_
k_T_		0.07		0.07		0.07				
k_P_		0.10		0.10		0.10				
k_H_		0.10		0.10		0.10				
Charge measurements **reference field**	0.03		0.03		0.03		0.03		0.03	
linac output constancy	0.01		0.01		0.01		0.01		0.01	
k_s_ (recombination)		0.14		0.14		0.14		0.14		0.14
kpol (polarity)		0.02		0.02		0.02		0.02		0.02
**Charge measurements in small field**	0.03		0.03		0.03		0.03		0.03	
k_small_ (microD/unshielded diode)		0.30		0.50		0.50		0.64		0.80
k_small_ (shielded diode)		0.30		0.60		0.70		1.00		
k_small_ (micro IC)		0.40		0.60		1.10		2.00		3.20
k_small_ (mini IC)		0.40		1.20		2.50		3.60		
**CAX positioning**										
microD/unshielded diodes	0.28		0.45		0.63		0.63		0.98	
shielded diodes	0.14		0.28		0.54					
micro IC	0.42		0.58		0.67					
mini IC	0.10		0.10		0.40					
**K_small_ error due to FWHM**										
microD/unshielded diode	0.03		0.03		0.02		0.03		0.03	
shielded diode	0.04		0.04		0.02		0.04		0.04	
mini IC	0.02		0.02		0.02		0.02		0.02	
micro IC	0.01		0.01		0.15		0.01		0.01	
**Quadratic summation**										
microD/unshielded diode	0.29	0.30	0.45	0.50	0.63	0.50	0.63	0.64	0.98	0.80
shielded diode	0.15	0.30	0.29	0.60	0.54	0.70		1.00		
micro IC	0.42	0.45	0.58	0.64	0.69	1.12		2.01		3.21
mini IC	0.11	0.45	0.11	1.22	0.40	2.51				
**Combined relative standard uncertainty**										
microD/unshielded diode	0.41		0.67		0.81		0.90		1.27	
shielded diode	0.34		0.66		0.89					
micro IC	0.62		0.86		1.31					
mini IC	0.47		1.22		2.54					

**Table 6:** The relative standard uncertainty for all components in the measurement of square fields.

Quantity	1 cm × 1 cm		1.5 cm × 1.5 cm		2 cm × 2 cm		2.5 cm × 2.5 cm		3 cm × 3 cm		4 cm × 4 cm		6 cm × 6 cm	
	RS uncert.		RS uncert.		RS uncert.		RS uncert.		RS uncert.		RS uncert.		RS uncert.	
	100 u_iA_	100 u_iB_	100 u_iA_	100 u_iB_	100 u_iA_	100 u_iB_	100 u_iA_	100 u_iB_	100 u_iA_	100 u_iB_	100 u_iA_	100 u_iB_	100 u_iA_	100 u_iB_
k_T_		0.07		0.07		0.07		0.07		0.07		0.07		0.07
k_P_		0.10		0.10		0.10		0.10		0.10		0.10		0.10
k_H_		0.10		0.10		0.10		0.10		0.10		0.10		0.10
Charge measurements reference field	0.02		0.02		0.02		0.02		0.02		0.02		0.02	
linac output constancy	0.01		0.01		0.01		0.01		0.01		0.01		0.01	
k_s_ (recombination)		0.14		0.14		0.14		0.14		0.14		0.14		0.14
kpol (polarity)		0.02		0.02		0.02		0.02		0.02		0.02		0.02
Charge measurements in small field	0.02		0.02		0.02		0.02		0.02		0.02		0.02	
k_small_ (microD/unshielded diode)		0.50		0.50		0.40		0.40		0.40		0.30		0.30
k_small_ (shielded diode)		0.70		0.60		0.50		0.40		0.40		0.40		0.30
k_small_ (micro IC)		1.10		0.60		0.40		0.40		0.40		0.40		0.30
k_small_ (mini IC)		2.50		1.20		0.70		0.50		0.40		0.40		0.30
CAX positioning														
microD/unshielded diodes	0.60		0.50		0.50		0.40		0.30		0.30		0.30	
shielded diodes	0.54		0.28		0.28		0.21		0.14		0.14		0.14	
micro IC	0.67		0.67		0.58		0.50		0.50		0.42		0.42	
mini IC	0.36		0.14		0.14		0.14		0.14		0.14		0.14	
K_small_ error due to FWHM														
microD/unshielded diode	0.02		0.03		0.03		0.03		0.03		0.03		0.03	
shielded diode	0.02		0.04		0.04		0.04		0.04		0.04		0.04	
mini IC	0.02		0.02		0.02		0.02		0.02		0.02		0.02	
micro IC	0.15		0.01		0.01		0.01		0.01		0.01		0.01	
Quadratic summation														
microD/unshielded diode	0.60	0.50	0.50	0.50	0.50	0.40	0.40	0.40	0.30	0.40	0.30	0.30	0.30	0.30
shielded diode	0.54	0.70	0.28	0.60	0.28	0.50	0.22	0.40	0.15	0.40	0.15	0.40	0.15	0.30
micro IC	0.69	1.12	0.67	0.64	0.58	0.45	0.50	0.45	0.50	0.45	0.42	0.45	0.42	0.37
mini IC	0.36	2.51	0.14	1.21	0.14	0.71	0.14	0.54	0.14	0.45	0.14	0.45	0.14	0.37
Combined relative Standard uncertainty														
microD/unshielded diode	0.78		0.71		0.64		0.57		0.50		0.43		0.43	
shielded diode	0.88		0.66		0.58		0.45		0.43		0.43		0.33	
micro IC	1.31		0.92		0.74		0.67		0.67		0.62		0.56	
mini IC	2.53		1.22		0.73		0.56		0.47		0.47		0.39	

The “grey” part of the table is for ionization chambers only, with k_T_, k_P_ and k_H_ listed as a type B uncertainties. The “blue” section of the table refers to uncertainty measurements related to the reference fields; 3 cm × 3 cm and 6 cm × 6 cm for cones and 10 cm × 10 cm for square fields. For this part k_s_ (recombination) was determined using ACDS data for Farmer chambers, which has a 0.14% uncertainty. Similarly, the uncertainty in the polarity correction (k_pol_) was determined as 0.02% (using ACDS data for Farmer chambers with a 20% uncertainty in the polarity correction). Both values are included in the summation for the micro and mini ionization chambers, and do not apply to solid state detectors. The linac output constancy was quantified by repeatedly measuring the reference field during a set of measurements (typically within a two h period) and was found to be 0.01%.

The “yellow” section of the table refers to the uncertainties for the small field measurements. The statistical uncertainty in the charge measurements in the small field (type A) is the maximum relative standard uncertainty for all different field sizes measured. Whereas, the k_small_ values (a term we derived) are a type B uncertainty, taken directly from the TRS‐483 protocol, Table 37, and is the uncertainties in the output correction factors (k values) listed.

The maximum acceptable central axis (CAX) positioning error was 0.3 mm (and was based on ACDS level 1‐B audits, where a 0.3 mm positioning limit is applied to accommodate sites with older water tanks and lower positional accuracy). Although IBA states a positional reproducibility of ±0.1 mm for the Blue Phantom2 tank, this was not used. The dosimetric error for each detector type at 0.3 mm was quantified using the scanned in‐line and cross‐line profiles for each detector.

The k_small_ error due to the measurement of the full width half maximum (FWHM), refers to the reproducibility for each square field and cone field size. The error in the field size measurements will contribute to error in the selection of the “k” value from Table 26 of TRS‐483.^1^

**Figure 8:**
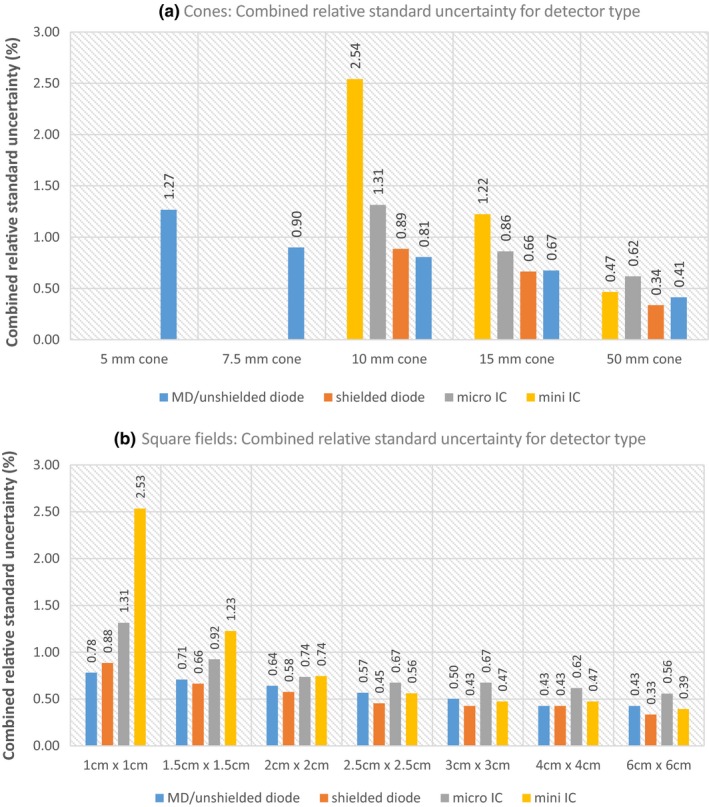
The combined relative standard uncertainty in the output factor measurements for all detector types investigated; MicroDiamond/unshielded diodes, shielded diodes, micro ionization chamber and mini ionization chambers for circular (a) and square fields (b). See also Tables 5 and 6.

Quadradic summation of Type A and Type B uncertainties was completed for each detector in the “purple” section. Both types of uncertainty were finally combined in quadrature to give the total relative standard uncertainty in the “green” section, and are shown in Fig. 8.

**Figure 9:**
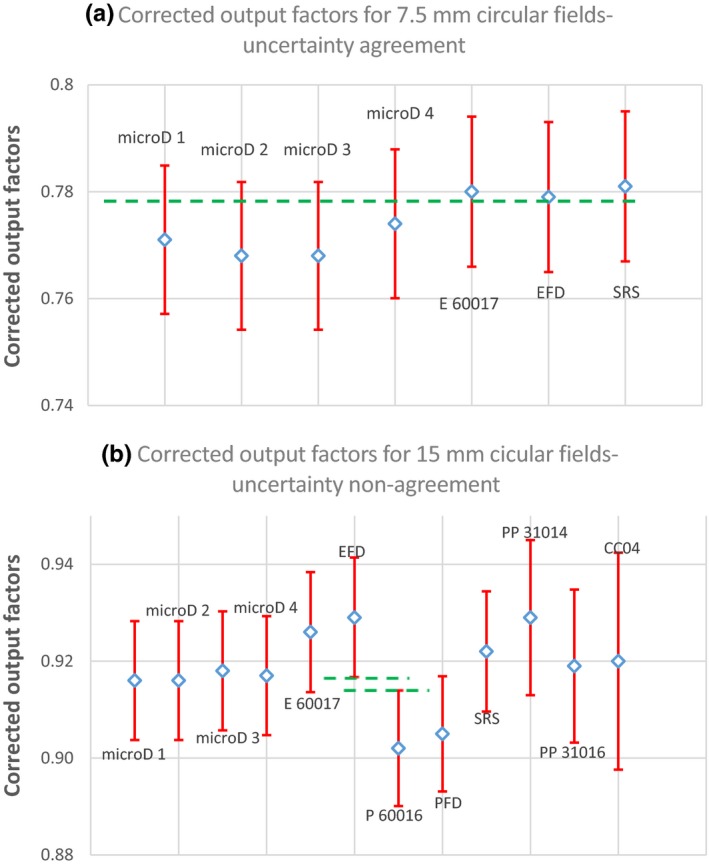
Plots of the output factors at the indicated circular field size are shown with horizontal displacement to allow each individual detector to be easily identified. Agreement within uncertainties (a) is shown for the 7.5 mm circular field size by the dashed green line passing through all error bars, and was seen for all detectors and field sizes examined except for the 15 mm circular field size (b), which was the single case of nonagreement. The maximum difference between detectors is shown by the green dashed lines.

The expanded uncertainty (k = 2) error bars for each detector type for all nominal field sizes examined were applied [Figs 3(b) and 4(b)]. Overall agreement was found for all detectors in all fields except for the 15 mm circular field [Fig 9(b)]. An example of agreement [Fig 9(a)], and the one case of nonagreement [Fig 9(b)] are shown in Fig. 9 with the results horizontally displaced to allow comparison of each result, as individual results are difficult to distinguish in [Figs 3(b) and 4(b)].

Agreement within uncertainties (a) is shown for the 7.5 mm circular field size by the dashed green line passing through all error bars, and was seen for all detectors and field sizes examined except for the 15 mm circular field size (b), which was the single case of nonagreement. The maximum difference between detectors is shown by the green dashed lines.

## 4 DISCUSSION

### 4.1 Corrected and uncorrected output factor values

Variations in the uncorrected output factors compared to the corrected output factors (with the TRS‐483 corrections factors applied) were reduced for all detectors investigated, particularly for the smaller circular fields (5–15 mm) and square field sizes (1 cm × 1 cm to 4 cm × 4 cm) (Figs 3, 4, 5, 6). This is most evident for the Pinpoint 3D PTW 31016 and IBA CC 04 detectors, which showed the largest variations in responses for the uncorrected output factors (circular and square fields) relative to the PTW 60019 microDiamond (Figs 5 and 6). For the 10 mm cone [Fig. 5(a)] the uncorrected values differed by 5.4 and 5.2% (PTW 31016 and CC04), and after correction reduced to 0.4 and 0.0% respectively. Similarly, for the square field results, both detectors yielded differences of 5.6 and 4.8% (PTW 31016 and CC04, uncorrected 1 cm × 1 cm) relative to the PTW 60019 microDiamond detector, which reduced to 2.2% (for both detectors) when corrected.

Interestingly, the uncorrected output factors for circular fields [Fig. 5(b)] measured with the two photon diodes, IBA‐PFD3G and PTW 60016, showed better agreement for the 15 mm cone field than the corrected values, and are slightly clearer in [Fig. 5(a) than Fig. 3(a)]. The output factor values when uncorrected were 0.930 (PTW 60016) and 0.928 (IBA‐PFD3G), which were slightly higher compared to the other detectors, but reduced to 0.902 and 0.905 respectively when corrected, which is well below the average corrected output factor (0.921) for all other detectors examined at that cone size [Fig. 3(b)]. This is also evident in the comparisons to the microDiamond detector for both circular and square fields (Figs 5 and 6). For the 15 mm corrected circular field results relative to the microDiamond [Fig. 5(b)], the difference in output factor doubled for each detector; photon‐diode PTW 60016 0.8% (uncorrected) to 1.6% (corrected) and for IBA‐PFD3G 0.6% (uncorrected) to 1.2% (corrected). This difference was clearer for the square field sizes; 1 cm × 1 cm to 3 cm × 3 cm relative to the PTW 60019 microDiamond (Fig. 6), with the uncorrected PTW 60016 values difference ranging between 0.4 and 0.1% and increasing to 1.6–0.4% when corrected. Similarly, the IBA‐PFD3G uncorrected differences (relative to the PTW 60019 microDiamond) were between 0.0 and 0.8%, and increased to 1.5–0.8% after correction [Fig. 6(b)]. All other results for cones and square fields showed an improvement when the protocols correction factors were applied.

Reasons for this difference with the photon diodes could be attributed to individual detector differences or could be related to the fact that both diodes are shielded, unlike the other diodes investigated in this work and thus have more uncertainty in k_s_ (Table 1). Shielded diodes are known to overestimate dose relative to water,^17^ and exhibit greater perturbations in small field measurements due to the additional metallic layer present around the active volume. Higher uncertainties in the correction factor are caused by the attenuation of low‐energy photons, and simultaneous increase in contributions from electron scatter.^2^, ^17^, ^18^, ^19^ These effects contribute to make larger and more uncertain correction factors.

The uncorrected output factor values measured in this work agree with previous work by Godson^12^ who used the IBA EFD, IBA PFD, and PTW 60018 SRS diodes and Pinpoint PTW 31014 ionization chamber. Cone diameters ranging between 10 and 40 mm (in 5 mm increments) and field sizes between 1 cm × 1 cm and 10 cm × 10 cm (in 1 cm × 1 cm increments) were examined, at the same SSD and depth as this investigation. Overall Godson^12^ reported reasonable consistency in uncorrected output factors (for all detectors) when using field sizes ≥2 cm × 2 cm. Whilst no correction factors were applied to the results, it was reported for smaller cone and field sizes that the IBA PFD diode over‐estimated the output factors, as occurred in this study. These results confirm that larger differences between detectors occur when using smaller field and cone sizes, and that shielded diodes have higher uncertainties due to their construction (namely due to the extra metallic layer).

This work also agrees with the findings by Shukaili,^11^ where the TRS‐483 protocol correction factors were applied to the IBA‐SFD detector used for stereotactic cone measurements (5–40 mm), and compared with EBT3 film and a DUO detector (an in‐house design consisting of two silicon diode arrays*).* Differences in detector and film responses reduced from 5.7% to 2% for the 5 mm cone, with an average agreement of ±0.8% for all 12 cone sizes investigated (after correction). Shukiali,^11^ refers to the recent AAPM practice guidelines, which recommends SRS‐SBRT annual QA for output factor tolerance is ±2% from baseline for >1.0 cm apertures and ±5% from baseline for ≤1.0 cm apertures.^20^ The combined relative standard uncertainty values determined for all detector types in this work (Tables 5 and 6, Fig. 8) are within these limits; ≤1.31% for all cone and square field sizes examined, except for the mini ionization chamber (CC04) measurement, which was; 2.54% (10 mm cone) and 2.53% (1 cm × 1 cm square field) (Fig. 8).

In general, the RSD values for the corrected output factors reduced (for all detectors) (Tables 2 and 3), with the percentage difference between the corrected and uncorrected values decreasing with increasing cone and field size. The largest reduction in RSD was for the 10 mm cone; 1.7% (uncorrected; 2.3%, corrected 0.6%) (Table 2), with all other RSD corrected values ≤1.2%. For the square fields the greatest difference in the RSD for the corrected and uncorrected values was 1.6%; 1 cm × 1 cm field (uncorrected; 2.3%, corrected 0.8%) (Table 3), with all other corrected RSD values being ≤0.6%. Two exceptions to the decrease in the corrected RSD were seen for the 15 and 50 mm cone results. The 15 mm cone showed no difference in RSD when corrected, and is likely due to the two photon diodes (PTW 60016 and IBA‐PFD) overcorrecting after the correction factors were applied as has been discussed. While for the 50 mm cone a 0.1% increase in the corrected RSD occurred, which was not evident elsewhere (uncorrected; 0.2%, corrected 0.3%), (Table 2).

### 4.2 PTW 60019 microDiamond intracomparison for the stereotactic cone output factor values

Four PTW 60019 microDiamond detectors were used to measure the output factors for the stereotactic cones (5, 7.5, 10 and 50 mm) and compared to investigate intra detector variations (Fig. 7, Table 4). The average of six measurements for each detector showed good agreement overall, with the 5 mm cone yielding the largest variation (1.4%), with an RSD of 0.7% (Table 4). The remaining cone sizes differed by ≤0.4% (RSD ≤ 0.3%), and agrees with the fact that smaller sized cones are known to yield higher variations in their output factor response and dosimetric characteristics.^11^, ^21^ Interestingly, the results for the 5 mm cones showed a difference in output factors which may be related to the detectors production date. Two microDiamond detectors with 123000 serial numbers produced slightly lower values of 0.683 and 0.680, while the 122000 serial numbered detectors yielded values of 0.690 and 0.691. As this difference was only evident for the 5 mm cone it might be worth future investigation as its significance is unknown.

### 4.3 The small field uncertainty budget

Lastly, the uncertainty budget for the measurement of small field output factors was determined and its components quantified (Tables 5 and 6). The combined relative standard uncertainties are summarized for both circular and square fields (Fig. 8), and the expanded uncertainty (k = 2) applied as error bars to Figs 3(b) and 4(b) and Fig. 9. The largest sources of uncertainty were; k_small_ (which is the uncertainties in the output correction factors stated in the protocol) and ranged between 0.30–3.60% for circular fields (Table 5) and 0.30–2.50% for square fields (Table 6). Tolabin^22^ also reported this in their small field uncertainty budget for square fields (0.5 cm × 0.5 cm to 4 cm × 4 cm) using two mini‐ionization chambers (IBA CC13, CC01), one micro‐ionization chamber (Razor NanoChamber) and one unshielded diode (IBA Razor). The other main source of uncertainty in our budget was the CAX positioning error (0.3 mm), which was determined using the in‐line and cross‐line profiles for each detector type scanned at each field or cone size. Its magnitude for each detector type ranged between 0.10–0.98% for circular fields (Table 5) and 0.14–0.67% for square fields (Table 6). Interestingly, Tolabin^22^ estimated this uncertainty (called µ_scan_) as much lower with a range between 0.001 and 0.012% uncertainty for all four detector types investigated. As Tolabin^22^ does not state the CAX positioning limit, this difference could be due to applying a smaller positioning error (0.1 mm), which would yield lower uncertainties particularly for steep gradient profiles.

Overall the uncertainties increased as the field or cone size decreased (Fig. 8) as was expected, and in general solid state detectors yield smaller uncertainties compared to the micro and mini‐ ionization chambers (10–50 mm circular fields and all square field sizes) (Fig. 8), which agrees with the budget prepared by Tolabin.^22^ This is most evident for the 1 cm × 1 cm square fields with the microDiamond/unshielded diodes having a combined relative uncertainty of 0.78%, shielded diodes 0.88%, while the micro and mini‐ionization chambers yield 1.31 and 2.53%, respectively.

The consistency between different detectors was assessed by examining all detector output factor measurements at each circular and square field size (Tables 5 and 6, Fig 9) after applying the expanded uncertainty to each result. All circular and square field size output factor measurements showed agreement within uncertainties [Fig 9(a)], except for the 15 mm circular field [Fig 9(b)]. The magnitude of the disagreement was small (0.003 between the IBA‐EFD3G minimum of 0.917 and 0.914 for the PTW 60016 maximum), and is interesting as the PTW 60016 uncorrected output factor value (0.930) for the 15 mm circular field had shown better agreement with the other corrected detector values (Section 4A), and would have yielded full agreement. The relatively large correction factor for this detector in this field contributes to a larger uncertainty in the correction factor for this detector. For agreement within uncertainties to occur between the two detector types (IBA‐EFD3G and PTW 60016) the uncertainty for either detector would need to increase by a factor of 1.25 (25%). Based on the good agreement for each field size investigated, the uncertainty budget appears to estimate the magnitude of the uncertainties reasonably well, and that the uncertainties stated for the correction factors in the protocol^1^ are appropriate, however based on our results the uncertainties for the PTW 60016 (and maybe the IBA‐PFD3G) have been underestimated.

## 5 CONCLUSIONS

The correction factors stated in the new IAEA TRS 483 protocol^1^ were applied to nine detectors used to measure the output factors in small square and circular fields. The corrected output factors showed reduced variations and were more consistent when compared to the uncorrected output factors. Both corrected output factors for the photon diodes (PTW 60016 and IBA‐PFD3G) seemed to overcorrect, respectively for the 15 mm cone and further investigation is needed to determine if this is due to individual detector responses or the correction factors in the protocol. Comparative investigations of the four PTW 60019 microDiamond detectors showed good overall agreement, however a slight difference in response for the smallest circular field (5 mm) was evident, and is likely indicative of the difficulties in measuring smaller fields. Lastly, the combined relative standard uncertainties for small field measurements were examined (≤2.54% for all detector types and all field sizes). Applying the expanded uncertainties to the results showed complete agreement for all detectors in all fields except for the 15 mm circular field where one minor disagreement was observed. This would suggest that the stated uncertainties and their magnitudes including for the correction factors stated in the protocol are appropriate. Overall this work suggests that by following the protocol’s procedures reliable and consistent output factor measurements can be made.

## CONFLICT OF INTEREST

None.
